# Efficacy of a glucose meter connected to a mobile app on glycemic control and adherence to self-care tasks in patients with T1DM and LADA: a parallel-group, open-label, clinical treatment trial

**DOI:** 10.20945/2359-3997000000334

**Published:** 2021-02-25

**Authors:** Felipe Martins de Oliveira, Luís Eduardo Procópio Calliari, Cecília Kauffman Rutenberg Feder, Maria Fernanda Ozorio de Almeida, Mariana Vilela Pereira, Maria Thereza Teixeira de Almeida Fagundes Alves, Sônia Aparecida Dias Garcia, Ligia Dinara Donizeti Reis, João Eduardo Nunes Salles

**Affiliations:** 1 Associação de Diabetes de Ourinhos Ourinhos SP Brasil Associação de Diabetes de Ourinhos (ADO), Ourinhos, SP, Brasil; 2 Irmandade Santa Casa de Misericórdia de São Paulo São Paulo SP Brasil Irmandade Santa Casa de Misericórdia de São Paulo, São Paulo, SP, Brasil

**Keywords:** Diabetes mellitus, type 1, glycated hemoglobin A, treatment adherence and compliance, self-management, blood glucose

## Abstract

**Objective:**

The main aim of the study was to evaluate the patients’ glycemic control and adherence to self-care tasks.

**Materials and methods:**

Patients with type 1 diabetes mellitus (T1DM) or latent autoimmune diabetes of the adult (LADA) using a multiple daily injection (MDI) regimen with carbohydrate counting (n = 25, Subgroup B) or fixed insulin dose (n = 25, Subgroup C) were allocated to use the application (app) for 12 weeks. Both subgroups were compared with each other and against a control group (n = 25, Group A) comprising patients with T1DM or LADA treated with continuous subcutaneous insulin infusion (CSII) in a parallel-group, open-label, clinical treatment trial. All patients had glycated hemoglobin (A1C) levels measured and were asked to fill out the Diabetes Self-Management Profile (DSMP) questionnaire at study start and end. The patients were instructed to measure capillary glucose six times daily in study weeks 4, 8, and 12.

**Results:**

Mean A1C levels decreased 0.725% in Subgroup C in intragroup analysis (p = 0.0063), and had a mean variation of 0.834% compared with Group A (p = 0.003). Mean DSMP scores increased 5.77 points in Subgroup B in intragroup analysis (p = 0.0004) and increased by a mean of 6.815 points in relation to Group A (p = 0.002).

**Conclusion:**

OneTouch Reveal improved both A1C levels and DSMP scores in patients with T1DM or LADA compared with standard treatment (CSII).

## INTRODUCTION

A 2019 projection from the International Diabetes Federation (IDF) ranked Brazil third among all countries in highest number of cases of type 1 diabetes mellitus (T1DM), with an estimated prevalence of 51,500 cases among children and adolescents (1). Concerningly, only 13.2% of all patients with T1DM have glycated hemoglobin (A1C) levels within target goals (2). Complications of T1DM can be prevented by strict patient adherence to self-care tasks and achievement of intensive glycemic control (3).

Adherence can be defined as the extent to which a person's behavior corresponds with the agreed recommendations from a health care provider (4). Adherence to treatment of chronic diseases is lower than 50% (5–8) and requires three additional medical visits per patient per year, increasing the average annual cost of individual treatment by $2,000 (6).

For optimal adherence in diabetes, the patients must follow difficult treatment regimens. For example, patients with T1DM and some of those with latent autoimmune diabetes of the adult (LADA) must be treated with continuous subcutaneous insulin infusion (CSII) or with multiple daily injection (MDI) regimens of fixed insulin doses or insulin doses conditioned to carbohydrate counting (9).

The Diabetes Self-Management Profile (DSMP) questionnaire is a self-reported measure of adherence (10) (Appendix 1) that has been translated into Brazilian Portuguese and validated for the Brazilian population by Teló and cols. (2). Questions in the DSMP contemplate different aspects of diabetes self-management, covering five essential self-care domains, namely, exercise, hypoglycemia, diet, blood glucose monitoring, and insulin (11,12).

Regular physical activity has been associated with a reduced risk of cardiovascular disease in patients with diabetes, decreasing all-cause mortality (13,14). However, insulin therapy in these patients must be managed with special care to reduce the risk of exercise-related hypoglycemia.

Regarding MDI regimens, the use of modern ultra–long-acting insulin analogues along with carbohydrate counting help reduce the risk of hypoglycemia; patients on CSII therapy also have important tools to prevent this complication (9,15).

In terms of diet, carbohydrate counting is currently considered the gold standard for estimation of meal-time insulin dose in patients with T1DM or LADA (9). Modern CSII has integrated bolus calculators that automatically determine how much insulin should be administered based on food intake (9).

The Brazilian Diabetes Society (SBD) recommends 4–8 capillary self-monitoring blood glucose (SMBG) measurements daily for patients with T1DM or LADA (9). One of the various tools currently available for SMBG is the OneTouch Reveal (LifeScan, Wayne, PA, USA) mobile phone application (app). This app connects with the OneTouch Select Plus Flex (LifeScan) meter allowing patients to enter insulin doses, carbohydrate intake, and physical activity data, automatically transferring capillary glucose readings from the meter to the app. Synchronization between the app and the meter further allows patients to share the data with their physicians.

The Diabetes Association of Ourinhos (ADO) is a nongovernmental organization affiliated with the Juvenile Diabetes Association. All patients treated at ADO receive care covered by the Brazilian Unified Health System (SUS) and following the guidelines recommended by SBD (9). Carbohydrate counting is recommended to all patients with T1DM or LADA receiving care at ADO, but the effectiveness of this approach depends on the patient's commitment to the treatment. Those who fail to cope with the demands of carbohydrate counting are placed on an MDI regimen with fixed insulin dose. Obtaining a CSII device through SUS requires time; therefore, many patients at ADO follow carbohydrate counting while waiting for the device.

Patients with T1DM and LADA face significant challenges to meet the considerable demands of self-care management. Based on these considerations, this study focused on the glycemic control and adherence to self-care tasks of patients with T1DM or LADA treated with an MDI regimen using the OneTouch Reveal self-monitoring application versus standard treatment (CSII, control group).

## MATERIALS AND METHODS

A total of 75 patients receiving care at ADO were allocated in a parallel-group, open-label, clinical treatment trial with a control group. The intervention lasted 12 weeks.

### Eligibility criteria

The eligibility criteria included a diagnosis of T1DM or LADA for at least 1 year and exclusive use of insulin analogues. Patients on MDI with carbohydrate counting and those on CSII therapy were required to be on these treatment regimens for at least 3 months.

### Exclusion criteria

The exclusion criteria for the study were pregnancy, current use of a mobile phone app other than OneTouch Reveal, visual impairment preventing proper use of the devices (CSII or glucose meter/mobile phone app), and failure of delivery of insulin analogues or other supplies by governmental agencies.

### Sample size definition

A review of the medical data of patients registered at ADO in 2018 identified 127 patients meeting the eligibility criteria. Among these, 32 were receiving CSII therapy, while the remaining were on an MDI regimen (37 counting carbohydrates and 58 on a fixed insulin dose regimen).

**Figure 1:**
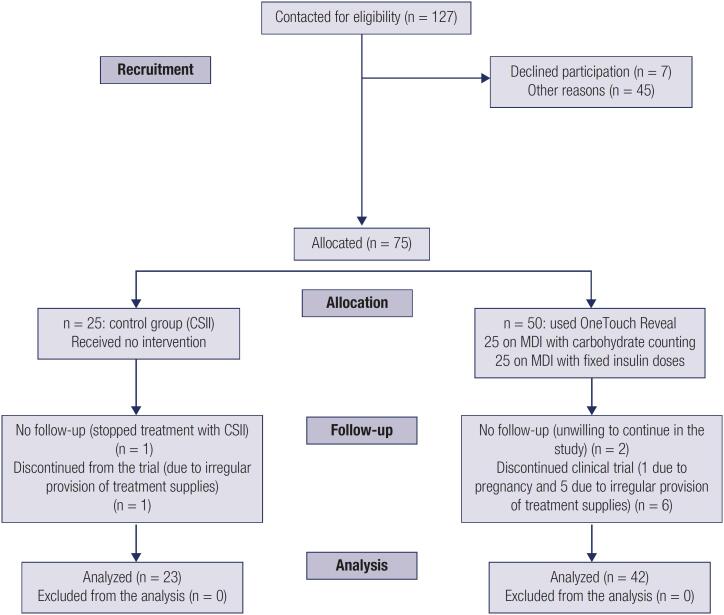
Flowchart of the study design

During the recruitment phase in January 2019, all 32 patients on CSII were invited to participate in the study, and the 25 patients who agreed comprised the control group. After selection of these patients, we recruited, following alphabetical order of patients who agreed to participate, the first 25 patients on MDI with carbohydrate counting and the first 25 patients on MDI with fixed insulin doses; these 50 patients comprised the intervention group (Figure 1).

The therapeutic regimen and type of CSII or insulin analogues used by the subjects were determined during routine follow-up appointments. Treatment regimens in place at the time of enrollment (CSII, MDI with carbohydrate counting, or MDI with fixed insulin doses) were established prior to patient recruitment during routine follow-up.

### Ethical aspects

This study was performed according to the ethical principles of medical research involving human subjects stated in the Declaration of Helsinki and approved by the Research Ethics Committee of *Irmandade Santa Casa de Misericórdia de São Paulo*. The study protocol was submitted electronically to *Plataforma Brasil* and received the Certificate of Presentation for Ethical Consideration (CAAE) under number 95031218.2.0000.5479. The protocol was also registered at the Brazilian Clinical Trial Registry (ReBEC) with the Universal Trial Number (UTN) U1111-1228-3527.

A free and informed consent form was signed by all participants older than 18 years. Legal guardians signed the consent forms for patients younger than 18 years, while these patients signed assent forms with age-appropriate language.

All expenses related to blood drawing and A1C tests performed at the study start and end, along with costs of meals and transportation for the participants at different stages of the research, were fully paid by the main researcher.

### Preintervention evaluation

Table 1 shows the baseline characteristics of the study participants. Preintervention assessments included measurement of A1C levels (by high-performance liquid chromatography) and evaluation with the DSMP questionnaire.

**Table 1:** Characteristics of the study sample (preintervention data)

Characteristics		Group A (n = 23)	Participants using OneTouch Reveal		
			Group B + C (n = 42)	Subgroup B (n = 22)	Subgroup C (n = 20)
Mean age (in years)		38.7	30.05	22.6	37.5
Sex, women/men (in %)		56.5/43.5	52.4/47.6	54.5/45.5	50/50
Mean age at diabetes diagnosis (in years)		20.7	17.3	11.3	23.3
Absolute number of patients with T1DM/LADA		19/4	38/4	22/0	16/4
Patients engaged in regular exercise (in %)		74	52.4	63.6	40
Mean weight (kg)		69.9	62.6	58.5	66.7
Mean body mass index (kg/m^2^)		25.7	23.7	22.8	24.6
Mean abdominal circumference (cm)		91.5	83.35	81.1	85.6
Patients according to education level (in %)	Incomplete elementary	4.34	9.52	4.54	15
	Complete elementary	None	7.14	9.09	5
	Incomplete secondary	None	4.76	4.54	5
	Complete secondary	43.4	42.8	45.4	40
	Incomplete higher	8.69	4.76	None	10
	Complete higher	34.78	26.2	31.81	20
	Postgraduate degree	8.69	4.76	4.54	5
Patients with microvascular complications (in %)	Retinopathy	13.04	11.9	4.54	15
	Nephropathy	13.04	14.3	None	30
	Neuropathy	17.39	19	9.1	30
Patients with positive diabetes autoantibodies (in %)	Anti-GAD	47.82	59.52	68.18	50
	Anti-islets	17.39	28.57	22.72	35
	Anti-insulin	13.04	11.9	13.63	10
Patients with other autoimmune diseases (in %)	Hashimoto's thyroiditis	39.13	30.95	45.45	15
	Celiac disease	4.34	None	None	None
Types of insulin used (in %)	Aspart	100	85.7	86.4	85
	Lispro	None	14.3	13.6	15
	Detemir	None	38.1	40.9	35
	U 100 Glargine	None	33.4	40.9	25
	Degludec	None	28.5	18.2	40
Mean duration of carbohydrate counting or CSII treatment (in months)		29.13	Not applicable	24.9	Not applicable
Patients with macrovascular complications (in %)		None			

Abbreviations: anti-GAD, anti-glutamic acid decarboxylase antibody; U 100 Glargine = 100 units of glargine insulin per mL of solution. Notes: For patients younger than 18 years, guardians with the highest level of education were considered. The same patient could present more than one concomitant microvascular complication. Subjects could have no positive autoantibody or more than one positive autoantibody. The analysis excluded patients without investigation of other autoimmune diseases at recruitment (for example, those with positive antibodies but no histological confirmation of celiac disease or atrophic gastritis). The same patient could present two or more concomitant autoimmune conditions.

### Patients’ allocation

Following the baseline evaluation, the patients were divided into the following groups (Figure 1):

- Group A: CSII therapy (no intervention, control group), n = 25.
- Subgroup B: MDI regimen with carbohydrate counting, n = 25.
- Subgroup C: MDI regimen with fixed insulin doses, n = 25.

The participants in Subgroups B and C were instructed to use the OneTouch Select Plus Flex meter and the OneTouch Reveal app for 12 weeks. The main researcher delivered the meters and instructed all 50 patients on MDI regimens on how to operate both meter and app. The first 3 weeks of the research were reserved for patients’ acquaintance with meter and app (run-in period).

Data from the devices were downloaded on study weeks 4, 8, and 12, and the patients were instructed to perform six capillary glucose measurements daily each week prior to these three time points. Each of these weeks was labeled a “focus week”.

In addition to the visits for data download, the participants maintained medical appointments with their usual endocrinologists at ADO throughout the study, and only data collected in the second focus week were evaluated in these appointments. Changes in insulin regimen implemented during these appointments followed SBD guidelines (9).

For ethical reasons, continuous glucose monitoring (CGM) data from Group A were only used for insulin adjustment and were not considered in the present analysis.

### Postintervention evaluation

Only data downloaded at focus weeks 4 and 12 were analyzed (Table 2). Blood drawing for A1C measurement and evaluation with the DSMP questionnaire were again performed at the end of week 12.

**Table 2:** Postintervention data (initial and final focus weeks)

Data				Group A (n = 23)	Participants using OneTouch Reveal		
					Group B + C (n = 42)	Subgroup B (n = 22)	Subgroup C (n = 20)
Mean frequency of daily capillary blood glucose measurements (in number of measurements)							
First FW				4.50	4.80	4.89	4.72
Third FW				4.67	4.67	4.66	4.68
(1)* p = 0.33, (2) p = 0.26, (3) p = 0.6, and (4) p = 0.62							
Mean blood glucose levels (mg/dL)							
First FW				171.89	171.01	177.60	164.42
Third FW				175.00	180.67	181.77	179.57
(1) p = 0.54, (2) p = 0.92, (3) p = 0.26, and (4) p = 0.42							
Mean postprandial blood glucose levels (mg/dL)							
First FW				176.01	180.13	188.00	172.27
Third FW				215.69	185.53	187.13	183.94
(1) p = 0.47, (2) p = 0.61, (3) p = 0.43, and (4) p = 0.76							
Mean total daily insulin dose (in units)							
First FW				44.40	54.55	56.30	52.80
Third FW				52.23	54.35	57.60	51.10
(1) p = 0.017, (2) p = 0.11, (3) p = 0.037, and (4) p = 0.33							
Mean daily dose of bolus insulin (in units)							
First FW				28.4	19.5	15.8	23.2
Third FW				27.9	18.9	17.4	20.5
(1) p = 0.94, (2) p = 0.38, (3) p = 0.42, and (4) p = 0.12							
Rates of hyperglycemia (in %)							
Total	First FW		39.27		38.49	40.40	36.29
	Third FW			42.32	44.39	45.18	43.47
(1) p = 0.71, (2) p = 0.66, (3) p = 0.86, and (4) p = 0.79							
Level 1	First FW			23.88	20.41	20.92	19.82
	Third FW			26.76	23.09	25.24	20.60
(1) p = 0.2, (2) p = 0.65, (3) p = 0.08, and (4) p = 0.13							
Level 2	First FW		15.38		18.08	19.47	16.46
	Third FW		15.55		21.30	19.94	22.87
(1) p = 0.19, (2) p = 0.36, (3) p = 0.17, and (4) p = 0.60							
Rates of hypoglycemia (in %)							
Total	First FW		4.36		8.66	6.81	10.51
	Third FW			3.79	8.20	6.33	10.07
(1) p = 0.009, (2) p = 0.09, (3) p = 0.004, and (4) p = 0.09							
Level 1	First FW		3.39		5.12	4.27	5.97
	Third FW		3.22		5.34	3.65	7.03
(1) p = 0.04, (2) p = 0.41, (3) p = 0.007, and (4) p = 0.03							
Level 2	First FW	0.97			3.54	2.55	4.54
	Third FW	0.57			2.86	2.69	3.04
(1) p = 0.02, (2) p = 0.06, (3) p = 0.02, and (4) p = 0.65							
Level 3	None of the patients presented level 3 hypoglycemia throughout the study						
Parameters of glycemic variability (% of patients within the indicated targets)							
Patients with more than 70% of all blood glucose levels within 70–180 mg/dL (in %)	First FW			35	14	18	10
	(1) p = 0.05, (2) p = 0.21, (3) p = 0.05, and (4) p = 0.45						
	Third FW			22	12	14	11
	(1) p = 0.31, (2) p = 0.47, (3) p = 0.33, and (4) p = 0.76						
Patients with a coefficient of variation of glucose levels below 36% (in %)	First FW			61	21	23	20
	(1) p = 0.0014, (2) p = 0.009, (3) p = 0.006, and (4) p = 0.83						
	Third FW			61	32	32	32
	(1) p = 0.02, (2) p = 0.05, (3) p = 0.06, and (4) p = 0.98						
Patients with less than 4% of hypoglycemia (in %)	First FW			16	15	9	6
	(1) p = 0.011, (2) p = 0.05, (3) p = 0.01, and (4) p = 0.53						
	Third FW			17	17	12	5
	(1) p = 0.012, (2) p = 0.17, (3) p = 0.002, and (4) p = 0.06						
Mean A1C levels (in %)							
Beginning of the study		7.28			8.21	7.76	8.67
End of the study		7.39			7.88	7.82	7.95
(1) p = 0.08, (2) p = 0.843, (3) p = 0.003, and (4) p = 0.009							
Mean DSMP score (in absolute points)							
Beginning of the study		53.43			45.02	47.04	43.00
End of the study		52.39			48.58	52.81	44.35
(1) p = 0.013, (2) p = 0.002, (3) p = 0.28, and (4) p = 0.04							

* P values of correlations between variations from the initial (first) to the final (third) focus week: (1) group A versus group B + C; (2) group A versus subgroup B; (3) group A versus subgroup C; (4) subgroup B versus subgroup C. Abbreviation: FW, focus week.

Since the DSMP results can be significantly biased by patients’ wish to please the research team (1,16), the questionnaires on both occasions were administered by staff members not assisting the patients.

### Primary outcome

The primary outcome was a statistically significant (p < 0.05) reduction in A1C levels between the initial and final evaluations, considering patients in Group B + C in intragroup analyses and compared with Group A.

### Secondary outcome

The secondary outcome was a statistically significant increase in DSMP scores between the initial and final evaluations considering patients in Group B + C in intragroup analyses and compared with Group A.

### Statistical analysis

The statistical analyses were performed using Excel 2016 (Microsoft, Seattle, WA, USA) and Action Stat, version 3.0 (Estatcamp, São Carlos, SP, Brazil).

Normality was tested with the Anderson-Darling, Kolmogorov-Smirnov, Shapiro-Wilk, and Ryan-Joiner tests. Differences between paired samples with normal distribution were analyzed with paired Student's *t* test. The z-proportion test compared proportions of success between the groups. Values were compared across different groups using independent samples *t* test. When the normality assumption was violated, we applied the Wilcoxon-Mann-Whitney test for independent samples, proportion test, or Wilcoxon test for paired data.

The independent samples *t* test, Wilcoxon rank sum test, and proportion test were used to analyze correlations between (1) Group B + C versus Group A, (2) Subgroup B versus Group A, (3) Subgroup C versus Group A, and (4) Subgroup B versus Subgroup C.

## RESULTS

Two patients in Subgroup C withdrew participation during the study. Eight other patients were excluded, including five for whom insulin analogues and/or supplies were not properly delivered by governmental agencies. Another patient in Subgroup C was excluded due to pregnancy occurring during the study. One patient in Subgroup B was excluded after receiving a CSII device by the governmental agency. Finally, another patient in Group A abandoned CSII during the study.

The final statistical analysis included the 65 remaining patients (Group A, n = 23; Subgroup B, n = 22; Subgroup C, n = 20) (Figure 1).

### Preintervention evaluation

Table 1 shows the baseline characteristics of the study sample.

### Postintervention evaluation

Table 2 shows postintervention data including the initial and final focus weeks. Group A compared with Group B + C showed a trend (p = 0.05) toward more patients with > 70% of blood glucose levels in the target range (70–180 mg/dL) in the first focus week. This finding no longer held true in the final focus week, when no differences were observed in the percentage of patients with > 70% of blood glucose levels within the target range in comparisons between Group A versus Group B + C (p = 0.31), Group A versus Subgroup B (p = 0.47), Group A versus Subgroup C (p = 0.33), or Subgroup B versus Subgroup C (p = 0.76).

### Primary outcome

**Figure 2:**
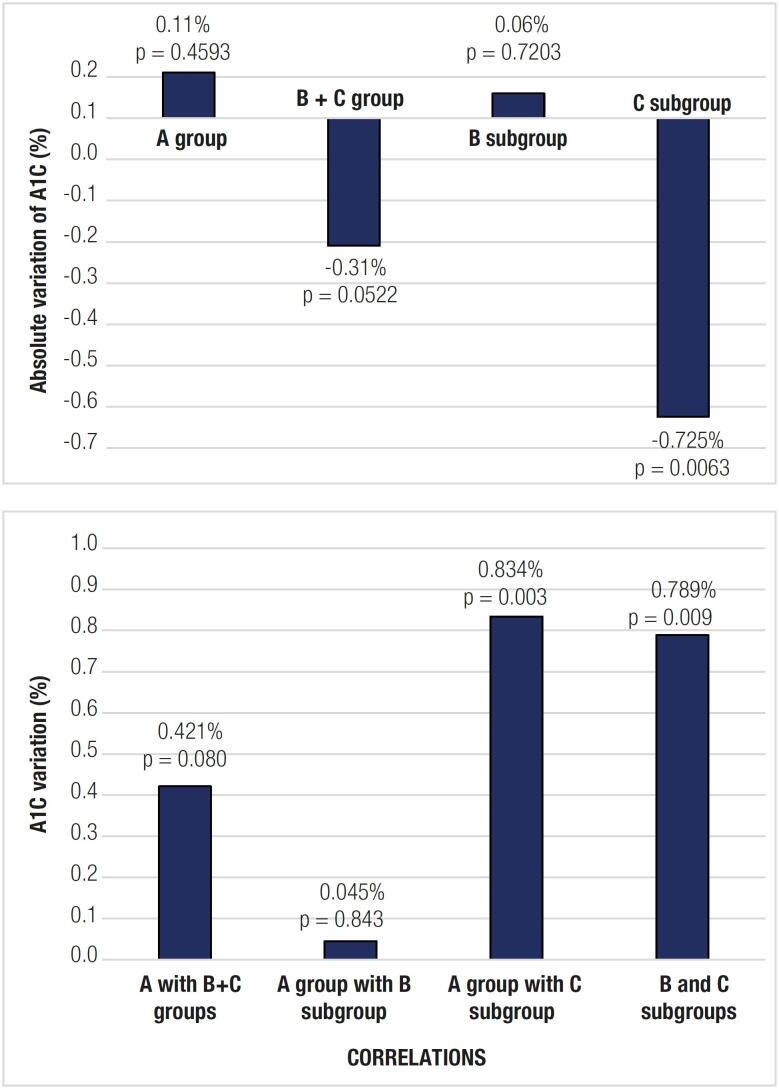
Primary outcome data The top graph shows absolute variations in A1C values from the beginning to the end of the study for each group and subgroup. The bottom graph shows correlations between different groups and subgroups based on variations in A1C values between the initial and final moment of the study.

Figure 2 shows the data related to the primary outcome. On intragroup analysis, the mean A1C level decreased by 0.725% in Subgroup C (p = 0.0063). A mean variation of 0.421% occurred between the initial and final A1C levels in Group B + C compared with Group A (p = 0.08). Similarly, no significant variation in A1C levels occurred when Subgroup B was compared with Group A (p = 0.843). In contrast, significant mean variations from initial to final A1C levels occurred between Subgroup C versus Group A (0.834%, p = 0.003) and Subgroup C versus Subgroup B (0.789%, p = 0.009).

### Secondary outcome

**Figure 3:**
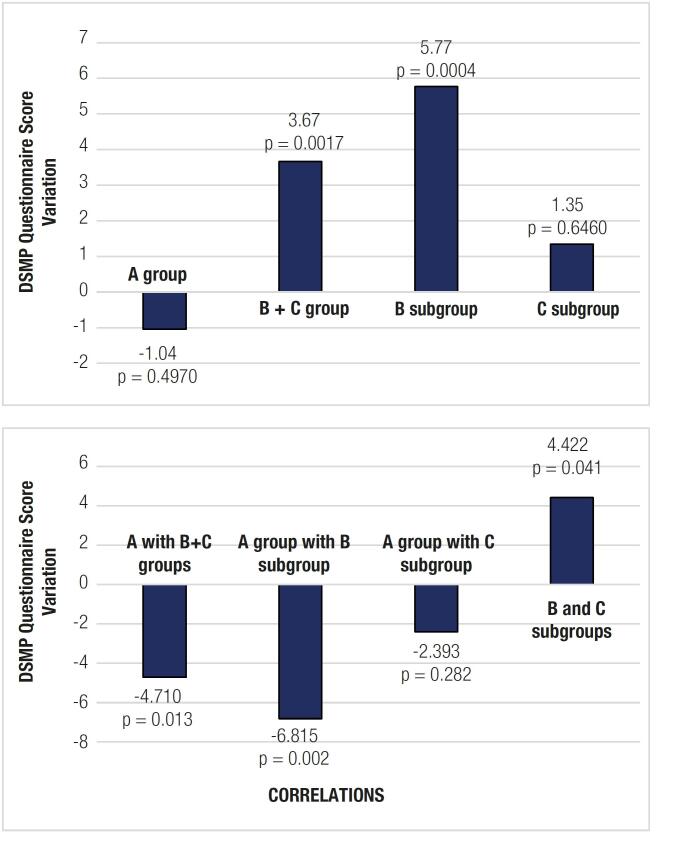
Secondary outcome data The top chart shows absolute variations between the DSMP scores from the beginning to the end of the study for each group and subgroup. The bottom chart shows correlations between different groups and subgroups based on variations in DSMP scores between the initial and final moment of the study.

Figure 3 shows the data related to the secondary outcome. On intragroup analysis, the variation in DSMP scores was not significant in Group A or Subgroup C, while the scores increased significantly in Subgroup B (mean 5.77 points, p = 0.0004). The DSMP scores increased from the initial to the final evaluation by a mean of 4.71 points in Group B + C compared with Group A (p = 0.013), by 6.815 points in Subgroup B compared with Group A (p = 0.002), and by 4.42 points in Subgroup B compared with Subgroup C (p = 0.041). No significant variations in DSMP scores occurred when Subgroup C was compared with Group A (p = 0.282).

## DISCUSSION

The present study showed that the use of a blood glucose meter synchronized to the OneTouch Reveal mobile phone app improved both A1C levels and DSMP scores in patients with T1DM or LADA compared with standard treatment (CSII).

This study is relevant within the Brazilian context as it identified a group of patients with diabetes receiving only insulin analogues. Another strength of the study is the unprecedented comparison of CSII as a control group versus another group using a glucose meter connected to a mobile phone app.

Even though the study was conducted prospectively, a potential limitation was the inclusion of patients from a single center. Other limitations include differences in action profile among the different types of insulin analogue used in the study and in CSII devices used by the participants in the control group.

Patient recruitment by alphabetical order and definition of the amount of time within the target range based on SMBG values are also potential limitations. Finally, the intervention in the present study lasted only 3 months, while Kirwan and cols. have reported better glycemic control with an intervention lasting 9 months (17).

Defining adherence can be complex in patients with T1DM or LADA (16), since it requires the assessment of tasks, behaviors (16–19), and treatment regimens that are not universal or static (16,19) in the absence of a reliable biological marker (e.g., measurement of medication levels) (16).

In assessing glycemic control, laboratory measurement of A1C alone is insufficient since the measurement may be negatively affected by interfering substances (16–17,19–20). In this sense, our analysis was based on CGM and SMBG results (16). In the first focus week, we observed a trend toward more patients in Group A maintaining capillary glucose at desirable levels (70–180 mg/dL) compared with those in Group B + C. The same finding was not confirmed in the final focus week, in which the percentage of patients with glucose within the target range at 70% of the time was comparable in Group A and Group B + C. However, this absence of difference between the groups could be explained by the small study sample.

### Primary outcome

The decrease in A1C levels between the initial and final analyses was especially significant in patients in Subgroup C compared with those in Group A. We are unable to evaluate the significance of this finding compared with the potential decrease in A1C levels produced by some oral antidiabetic drugs for type 2 diabetes (9).

We believe that the findings of our study reflect a better understanding of the disease and self-care strategies (21), as well as an interest in blood glucose reports and glucose diaries. These features have been mentioned in a meta-analysis by Boyle and cols. as the most desirable in mobile phone apps for patients with diabetes (22). Because OneTouch Reveal is only meant for documentation purposes and lacks a bolus calculator, the use of this app perhaps added very little to individuals on MDI with carbohydrate counting, probably explaining the absence of significant A1C variation in Subgroup C when compared with Group A.

Aligned with the favorable results found in the present study, a systematic review by Knox and cols. has shown that technology-based interventions increase the frequency of SMBG (18). Indeed, by correlating capillary blood glucose readings with information provided by adherence to self-care activities, the patients can establish cause-and-effect relationships and correct problems related to glycemic control commonly observed in clinical practice. Additionally, the chronological correlation between capillary blood glucose readings and events influencing the performance of self-care tasks can better guide the physician toward choosing specific therapies.

### Secondary outcome

Self-reported questionnaires may be useful as complementary information to objective assessments such as A1C and SMBG measurement (16). In the present study, DSMP scores correlated with adherence to self-care tasks (2,16), although the questionnaire items were not adaptable to the different treatment regimens (16). We found that the use of a glucose meter synchronized to the OneTouch Reveal mobile phone application improved the perception of self-care tasks in the subgroup of patients who already used insulin on MDI with carbohydrate counting regimen, despite the absence of A1C reduction. We postulate that the meter synchronized to the application enabled a more accurate view of all DSMP items by the patients, suggesting that the application can help patients on an MDI regimen to organize the daily demands involved in controlling their disease.

In conclusion, the use of mobile phone intervention apps synched to capillary blood glucose meters could play a key role in diabetes self-management in a country like Brazil, where CGM is still not widely established. The OneTouch Reveal app used in the present study improved both A1C levels and DSMP scores in patients with T1DM or LADA compared with standard treatment (CSII). In practice, patients on MDI with fixed insulin doses are likely to benefit most from this tool.

## Appendix 1 Diabetes Self-Management Profile (DSMP) questionnaire

Taking care of diabetes means doing a lot of different things like taking insulin, doing blood sugar tests, following a meal plan, getting exercise and dealing with low and high blood sugars. It is not easy doing all of these things exactly the way doctors and nurses might want. Very few patients with diabetes do everything exactly according to plan. Sometimes, there are other things that grab your attention or you might just forget to take care of your diabetes, even though you may have wanted to. Most patients with diabetes develop their own habits for taking care of their diabetes that are comfortable for them. What we are trying to learn in this interview is what you usually do to take care of your diabetes. I will ask you questions and write down your answers. You may stop me at any time, or go back to earlier questions to change your answers. Only your health team will see or hear your answers. Your participation is completely voluntary. You do not have to answer any question that you do not want to answer. You will not be penalized in any way if you decide to skip a question. Your answers won't be shared with anyone else, so you can feel comfortable telling me exactly what you do, not just what you think you are supposed to do or what you think I want you to say. So, try to be completely honest with me about what you have usually done in taking care of your diabetes in the past 3 months.

### EXERCISE

One part of taking care of diabetes is getting regular exercise, like running, bike riding, and swimming. Some patients manage to do this very regularly, while others have a hard time finding the time to get enough exercise. In this part of the interview, I'll be asking about your exercise habits. This could be something like taking part in sports, physical education or walking or riding your bike. Try to be as honest and accurate as you can about your exercise habits in the past 3 months.

1. What kind of exercise do you get? __________________________________________________________________
  In the past 3 months, how often have you gotten one of those kinds of exercise for at least 20 minutes?
  (4) More than three times per week.
  (3) 2-3 times per week.
  (1) Once a month.
  (0) Less than once per month.
2. If you get more exercise than usual, or if you plan to get more exercise, do you make changes in your diet or insulin? [If respondent replies “no”, circle 0 and skip to question 3]
  What do you do? __________________________________________________________________________________
  In the past 3 months, can you remember how many times you made this change?
  (4) I exercise so consistently that adjustments are unnecessary.
  (4) I always eat more or take less insulin.
  (3) I frequently eat more or take less insulin (2-3 times per week).
  (2) Sometimes I eat more or take less insulin (once a week).
  (1) I occasionally eat more or take less insulin (few times a month).
  (0) I eat less than usual or take more insulin or do not adjust eating or insulin.
3. If you get less exercise than usual, or if you plan to get less exercise, do you make changes in your meal plan or insulin? [If respondent replies “no”, circle 0 and skip to question 4]
  What do you do? __________________________________________________________________________________
  In the past 3 months, can you remember how many times you made this change?
  (4) I exercise so consistently that adjustments are unnecessary.
  (4) I always eat less or take more insulin.
  (3) I frequently eat less or take more insulin (2-3 times per week).
  (2) Sometimes I eat less or take more insulin (once a week).
  (1) I occasionally eat less or take more insulin (few times a month).
  (0) I eat more than usual or take less insulin or do not adjust eating or insulin.

### HYPOGLYCEMIA

Everyone with diabetes has low blood sugar reactions now and then that can lead you to feel dizzy, sweaty, shaky, confused, weak, hungry or irritable. Your doctor and nurses have probably taught you some things to do to keep low blood sugars from happening and to take care of yourself when they do happen. This part of the interview is about what you usually do about low blood sugar reactions. Try to be as honest and accurate as you can about what you did about low blood sugar in the past 3 months.

4. Do your keep something handy in case you have an insulin reaction or your sugar gets too low? For example, when you are at work or at a ball game, or in the car and your sugar gets too low, do you have something handy to eat?
  (1) Yes
  (0) No
5. If you think you have a low blood sugar, how often do you test before treating?
  (4) I always test before treating a low blood sugar or (insist that they have never had a low blood sugar).
  (3) I usually test before treating a low blood sugar (75% of the time) or (more than half the time).
  (2) Sometimes I test before treating a low blood sugar (50% of the time) or (half the time).
  (1) I infrequently test before treating a low blood sugar (25% of the time) or (less than half the time).
  (0) I never test before treating a low blood sugar.
6. People manage low blood sugar in many different ways. What do you usually do to treat your low blood sugar reactions? [If respondent replies he/she eats something, probe “How many grams of carbs is that?”] Then [If you take a specified amount of carbs not equal to 15 g, probe “Is that the amount your doctor told you to take?”] Then [If he/she takes a prescribed amount of carbs, but does not mention testing, probe “do you test after eating?”]
  (4) I’m careful to quickly take the prescribed amount of carbohydrates (15 g if applicable) and test blood if possible after 10 minutes or (insist that they have never had a low blood sugar).
  (3) I take prescribed amount of carbs and go on (do not test).
  (2) I take carbs (not the prescribed amount) without considering how much.
  (1) I continue treatment until symptoms go away.
  (0) I ignore symptoms until I get a chance to do something (waiting until it is convenient to treat symptoms).
7. Do you wear or carry anything that identifies you as having diabetes, like a card or bracelet?
  (2) I wear a necklace, bracelet or charm.
  (1) I carry billfold identification card only.
  (0) No diabetic identification readily available.

### EATING

Doctors, nurses and dieticians ask patients with diabetes to follow a meal plan that allows them to maintain a healthy weight and good blood sugar control. Lots of things can get in the way of doing this and, even when they try their best, many patients still struggle with eating exactly according to the plan. In this part of the interview, I'll be asking about your eating habits. Try to be as honest and accurate as you can about your eating habits in the past 3 months.

8. Do you measure your food, count carbs (or use exchanges) to figure out how much you should eat, or do you generally eat the same amount of food without counting carbs? [If respondent replies “I count carbs” ask “Tell me how you would count carbs for food you have never eaten before?”]
  (3) I use carb counting (or exchange list) as a guide and measure food or read labels.
  (2) I use carb counting (or exchange list) as a guide, but know meal plan well enough so that I can eat the right amount without measuring or reading labels.
  (1) I eat about the same amount of food each meal, but I do not measure or use carb counting (or exchange list).
  (0) I eat the amount I am hungry for and do not follow any set patterns of type or amount of foods.
9. There are foods that we all should avoid such as sweets and fatty foods like cookies, cakes, ice cream, chips, pizza, french fries, hot dogs, or others. Eating some of these foods is not necessarily bad for you; however, eating large amounts of sweets and/or fatty foods is not good for you. In the past 3 months, how often have you eaten more of these foods than is healthy?
  (4) Occasionally (few times a month or less).
  (3) Sometimes (once a week).
  (2) Frequently (2-3 times per week).
  (1) Almost always (4 or more times per week).
  (0) Once a day or more.
10. Sometimes people eat MORE food than what is on their meal plan. This does not include times when you should eat more when you get more exercise or when your sugar gets low. This might be when you eat because you are extra hungry or you might snack some before dinner. In the past 3 months, how often have you eaten more than what is recommended for your meal plan?
  (4) Never or hardly ever (1-2 times in the last 3 months).
  (3) Seldom (once a month).
  (2) Occasionally (few times each month).
  (1) Frequently (2-3 times per week).
  (0) Almost daily (4 or more times per week).
11. Before you eat MORE than you normally would, do you make any changes in your insulin? What do you do?
  (1) I take MORE insulin when I eat more.
  (0) I take LESS insulin when I eat more.
  (0) I do not adjust insulin.
12. Sometimes, people eat LESS food than what is on their diet plan. This does not include when your exercise changes, when you are sick or when your sugar is too high. This might be times when you just do not feel like eating everything on your plate. Before you eat LESS than you normally would, do you make any changes in your insulin? What do you do?
  (1) I take LESS insulin when I eat less.
  (0) I take MORE insulin when I eat less.
  (0) I do not adjust insulin.

### BLOOD GLUCOSE TESTING

Some patients do all of their blood sugar tests, but lots of other patients have trouble doing all of the tests their doctors and nurses want them to do. Next, I'll be asking about your habits when it comes to testing your blood sugar. Try to be as honest and accurate as you can about your testing habits in the past 3 months.

13. In the past 3 months, how often have you tested your blood sugar levels?
  (4) I test blood sugar 6 or more times daily.
  (3) I test blood sugar 4 or 5 times daily.
  (2) I test blood sugar 2 or 3 times daily.
  (1) I test blood sugar at least once daily.
  (0) I do not test or I test less than once a day.
14. How often has the doctor suggested that you test?
  (4) 6 or more times daily.
  (3) At least 4 or 5 times daily.
  (2) At least 2 or 3 times daily.
  (1) At least once daily.
  (0) I do not know.
15. How often do you test your blood sugar within 30 minutes before a meal?
  (4) I always test within 30 minutes before every meal.
  (3) I usually test within 30 minutes before meals (75% of the time) or (more than half the time).
  (2) Sometimes I test within 30 minutes before meals (50% of the time) or (half the time).
  (1) I infrequently test within 30 minutes before meals (25% of the time) or (less than half the time).
  (0) I never test within 30 minutes before meals.
16. How often do you test your blood sugar within 2-3 hours after a meal?
  (4) I test within 2-3 hours after a meal 4 or more times per week.
  (3) I test within 2-3 hours after a meal 3 times per week.
  (2) I test within 2-3 hours after a meal 2 times per week.
  (1) I test within 2-3 hours after a meal once a week.
  (0) I never test within 2-3 hours after meals.
17. How often do you test your blood sugar within 2-3 hours after heavy or intense exercise?
  (4) I always test within 2-3 hours after exercise.
  (3) I usually test within 2-3 hours after exercise (75% of the time) or (more than half the time).
  (2) Sometimes I test within 2-3 hours after exercise (50% of the time) or (half the time).
  (1) I infrequently test within 2-3 hours after exercise (25% of the time) or (less than half the time).
  (0) I never test within 2-3 hours after exercise.
18. In the past three months, how often have you adjusted your insulin dose, your diet or your exercise when your blood sugar test results were running high?
  (4) I made an adjustment every time it was needed.
  (3) I usually made an adjustment when needed (> 75%) or (more than half the time).
  (2) Sometimes I made an adjustment when needed (> 50%) or (half the time).
  (1) I infrequently made an adjustment when needed (< 50%) or (less than half the time).
  (0) I never made an adjustment.
19. Do you ever test for ketones?
  (1) Yes
  (0) No
  If you have two blood sugar results above 240 in a row, how often do you do a ketone test?
  (4) I can't remember having 2 consecutive blood sugar results above 240.
  (3) I always test after 2 consecutive blood sugar results above 240.
  (2) I usually test after 2 consecutive blood sugar results above 240.
  (1) I occasionally test after 2 consecutive blood sugar results above 240.
  (0) I never test after 2 consecutive blood sugar results above 240.
20. When you are sick, how often do you do a ketone test? [If respondent replies “always”, probe for number of times/day]
  (4) I always test several times a day when sick.
  (3) I always test once a day when sick.
  (2) I usually test once a day when sick.
  (1) I occasionally test when sick.
  (0) I never test when sick.

### INSULIN

Taking insulin shots includes measuring the doses carefully, taking the shots on time, and maybe changing the dose depending on your blood sugar test results. This is all very complicated and takes time that many patients would prefer to spend doing other things. This part of the interview is about what you usually do about your insulin shots. Try to be totally honest when you answer my questions.

21. In the last three months, how often have you delayed taking your insulin?
  (4) Never, I always take insulin on time.
  (3) I delayed once a month or less (1-3 times in the last 3 months).
  (2) I delayed once a week or less.
  (0) I delayed more than once a week.
22. In the past 3 months, how often have you taken MORE than the prescribed amount of insulin, even more than your sliding scale allows for?
  (4) I always took prescribed amount.
  (3) I took more than prescribed amount (1-3 times in the last 3 months).
  (2) I took more than prescribed amount (4-6 times in the last 3 months).
  (1) I took more than prescribed amount (7-10 times in the last 3 months).
  (0) I took more than prescribed amount (more than 10 times in the last 3 months).
23. In the past 3 months, how often have you taken LESS than the prescribed amount of insulin, even less than your sliding scale allows for?
  (4) I always took the prescribed amount.
  (3) I took less than prescribed amount (1-3 times in the last 3 months).
  (2) I took less than prescribed amount (4-6 times in the last 3 months).
  (1) I took less than prescribed amount (7-10 times in the last 3 months).
  (0) I took less than prescribed amount (more than 10 times in the last 3 months).
24. In the last three months, how often have you missed taking an insulin shot because you forgot or were too busy?
  (4) Never forgot, I always take insulin.
  (3) I forgot once a month or less (1-3 times in the last 3 months).
  (2) I forgot once a week or less.
  (0) I forgot more than once a week.
